# Enhancing the robustness of vision transformer defense against adversarial attacks based on squeeze-and-excitation module

**DOI:** 10.7717/peerj-cs.1197

**Published:** 2023-01-13

**Authors:** YouKang Chang, Hong Zhao, Weijie Wang

**Affiliations:** School of Computer and Communication, Lanzhou University of Technology, LanZhou, GanSu, China

**Keywords:** Adversarial attack, Defence against adversarial examples, Vision transformer, SE module

## Abstract

Vision Transformer (ViT) models have achieved good results in computer vision tasks, their performance has been shown to exceed that of convolutional neural networks (CNNs). However, the robustness of the ViT model has been less studied recently. To address this problem, we investigate the robustness of the ViT model in the face of adversarial attacks, and enhance the robustness of the model by introducing the ResNet- SE module, which acts on the Attention module of the ViT model. The Attention module not only learns edge and line information, but also can extract increasingly complex feature information; ResNet-SE module highlights the important information of each feature map and suppresses the minor information, which helps the model to perform the extraction of key features. The experimental results show that the accuracy of the proposed defense method is 19.812%, 17.083%, 18.802%, 21.490%, and 18.010% against Basic Iterative Method (BIM), C&W, DeepFool, DI^2^FGSM, and MDI^2^FGSM attacks, respectively. The defense method in this paper shows strong robustness compared with several other models.

## Introduction

Convolutional neural networks (CNNs) play an important role in artificial intelligence, such as in computer vision (CV) (Wu et al., 2022), natural language processing (NLP) (Messina et al., 2021) and speaker recognition (SR) (Xiao et al., 2022). However, researchers have recently pointed out that Transformer networks have made great progress in the field of NLP (Lauriola, Lavelli & Aiolli, 2022) by solving the long-range text association problem using the Attention mechanism compared to CNN networks. After that, researchers proposed network structures such as bidirectional encoder representations from transformers (BERT) (Kenton & Toutanova, 2019) and generative pre-training (GPT) (Radford et al., 2018) based on Transformer networks and achieved better results. Thanks to the successful application of Transformer in NLP, Dosovitskiy et al. (2020) proposed ViT model to apply Transformer structure in CV, compared with CNN model, ViT model and its variants showed better results in semantic segmentation (Strudel et al., 2021) medical image detection (Chen et al., 2022) and target detection (Alamri, Kalkan & Pugeault, 2021). With the continuous development of artificial intelligence, Transformer structure will be applied in more fields.

However, Goodfellow, Shlens & Szegedy (2014) pointed out that CNN structures are vulnerable to adversarial example attacks, and if small perturbations that are difficult for the human visual system to observe are added to the input data, the network model will output incorrect results with a high confidence rate, leading to a significant decrease in its robustness. Then, the researchers further showed that the phenomenon of adversarial attack not only exists in CNN models, but also ViT models are vulnerable to adversarial example attack, since ViT models have a tendency to surpass CNN models in terms of performance, how to safely deploy ViT models in practical applications has also become a focus of researchers.

In order to defend against the threats posed by adversarial attacks to artificial intelligence security applications, researchers have investigated multiple network models for adversarial attack defense methods, which at this stage are mainly divided into three categories: (1) data preprocessing for adversarial examples; (2) enhancing the robustness of deep neural networks; and (3) detecting adversarial examples. Data preprocessing methods such as denoising (Aneja et al., 2022; Xu et al., 2022) and data compression (Chang et al., 2022; Zhang, Yi & Sang, 2022). The advantages of these methods are faster computation and no need to modify the network structure, the disadvantages are that denoising and data compression can cause loss of information in the image, the neural network cannot extract features adequately, which makes the neural network make wrong judgments. Enhancing the robustness of deep neural networks improves the complexity of the network by increasing the stochasticity and cognitive performance of the network model, such as the deep compression network proposed by Gu & Rigazio (2014), defense distillation methods (Shao et al., 2022) and bio-inspired defense methods (Nayebi & Ganguli, 2017). Such methods require retraining the network, have high computational overhead, and remain less effective in defending against specific attacks that are carefully designed. The detection of adversarial examples defense method is to distinguish between adversarial examples and clean examples, if the detection is a clean example, it is fed into the neural network, and if the detection is an adversarial example, it is rejected to be fed into the neural network, common methods are generative adversarial network (GAN) (Esmaeilpour, Cardinal & Koerich, 2022) network based defense methods, MagNet (Meng & Chen, 2017) and other methods. However, the training process of using GAN network as a defense mechanism has a large overhead and its defense capability is not significantly improved if it is not trained properly; when MagNet is used as a defense method, it has good defense capability against black-box and gray-box attacks, but its performance is still low in the case of white-box attacks.

In summary, the above methods effectively improve the robustness of CNN models, but research on the robustness of ViT network structures is lacking. In order to deploy ViT model-based applications safely in real production life, the robustness of ViT models needs to be investigated. Based on this, this paper will explore the robustness of ViT models in the face of adversarial attacks. Firstly, ViT model learns fewer high-frequency features, which makes ViT more robust compared to other models; secondly, it was pointed out (Shao et al., 2021) that the robustness of the neural network models can be improved when the model is composed of Transformer structures and CNN modules. Therefore, the Squeeze-and-Excitation (SE) module is combined with the ResNet structure, the ViT structure is introduced together to propose the ResNet-SE-ViT model.

The main work of this article is as follows:

• The introduction of the ResNet-SE module into the ViT model to enhance the robustness of the network model in the face of adversarial attacks.
• The proposed method can effectively defend against white-box and black-box attacks.
• By comparing with the ViT model and its variants, the proposed model exhibits strong robustness.

## Related Work

The Transformer structure was originally proposed by Vaswani et al. (2017) and was mainly used in NLP tasks. Compared to recurrent neural network (RNN), the Transformer structure has achieved promising results. After that, Dosovitskiy et al. (2020) used the Transformer structure in the CV field and proposed the ViT model. In this section, several common ViT models and a study on the robustness of ViT models are introduced.

### ViTs and variants

Compared to the CNN models ResNet and EfficientNet, the ViT model achieves excellent performance on the large datasets ImageNet-21K and JFT-300M. The ViT model is comprised of three main components: an Embedding layer, a Transformer Encoder block and a Multilayer Perceptron (MLP) layer. The ViT model first splits an image into *P* ×* P* sized patches sequence, then flattened the patches sequence into 1D vector through linear projection, inserts the [CLS] token and Position Embedding into the Transformer Encoder block; in the Encoder block, the information of different regions learned is combined using the multi-head self-attention (MHSA) mechanism; finally, the MLP is used for image classification.

Yuan et al. (2021) found that ViT models cannot extract local details of images well and generate a large number of useless features during the training process, To address this problem, they proposed the tokens-to-token ViT (T2T-ViT) model. The patch embedding in the ViT model is replaced using the T2T structure, which progressively merges neighbouring tokens into a single token, extracting the local information by the surrounding tokens. Finally, the extracted information is fed into the ViT network for image classification. Han et al. (2021) proposed the Transformer-iN-Transformer (TNT) model. It mainly consists of stacked TNT blocks; each TNT block includes an outer Transformer block and an inner Transformer block. The outer Transformer block performs patch embedding on the image, the inner Transformer block extracts local features from the pixel embedding, then projects them into the patch embedding space through a linear transformation, which is added to the patch embedding. Wang et al. (2021) found that if high-resolution images are fed into ViT, it would take up high computational resources or even lead to overflow. To this end, they proposed the Pyramid Vision Transformer (PVT) model, which introduces a pyramid structure into the Transformer, continuously learning the generated multi-scale, high-resolution feature maps as the network structure deepens, and reducing computational effort by introducing spatial-reduction attention (SRA).

### Study of ViT model robustness

Due to the successful application of the ViT model and its variants in the CV field, researchers have started to focus on its robustness. Aldahdooh, Hamidouche & Deforges (2021) indicated that compared to the CNN model, the ViT variant can effectively defend against *L*_*p*_ parametrization and color channel perturbations (CCP) adversarial attacks, if the adversarial examples are again subjected to CCP adversarial attack, it can be remapped back to the clean example space; meanwhile, it is pointed out that adding attention blocks to the ViT model can effectively reduce the transferability of the CNN with the ViT model. Mahmood, Mahmood & VanDijk (2021) investigated the robustness of the ViT model against adversarial examples. They found that the ViT model was not secure against white-box attacks, such as C&W and APGD, only 6% accurate even against PGD and MIM attacks; subsequently, further research found that the adversarial examples were non-transferable between the Transformer and CNN, and proposed an integrated defence model that fusing the Transformer and CNN, which could not defend against white-box attacks, but was more robust against black-box attacks and did not reduce the accuracy of clean images. Mao et al. (2021) proposed the Robust Vision Transformer (RVT) method to effectively defend against the effects caused by adversarial attacks. The robustness of each module in the ViT model was also studied and analysed, they found that the model was less robust when the Transformer block in the model had a large spatial resolution; on the contrary, it helped to enhance the robustness of the model when the Transformer block gradually reduced the spatial resolution; the accuracy of clean images and adversarial examples both improved when the number of heads of the multi-head attention mechanism was increased to 8. This is because increasing the number of heads extracts attentional information from all aspects of the image, this complete, non-redundant attentional information introduces more visual relationships, thus improving the robustness of the model.

In summary, the current research on the robustness of the ViT model has shortcomings: firstly, most of the existing research analyzes the robustness of the modules in the ViT model and does not propose an effective defense method against adversarial attacks; secondly, the proposed defense method has poor generalization capability, that is, it can only defend a portion of the adversarial examples and cannot effectively cover both white-box and black-box attacks.

Based on the above reasons, this paper introduces the SE module into the ViT model and proposes the ResNet-SE-ViT model to defend against the impact of adversarial attacks. First, the ViT model can extract global features of images and learn less high-frequency information, which is slightly more robust than the CNN model; second, the SE module focuses on learning local features and effectively learns detailed information of textures and lines; finally, the proposed defense method can effectively defend against both white-box and black-box attacks.

## ResNet-SE-ViT

**Figure 1:**
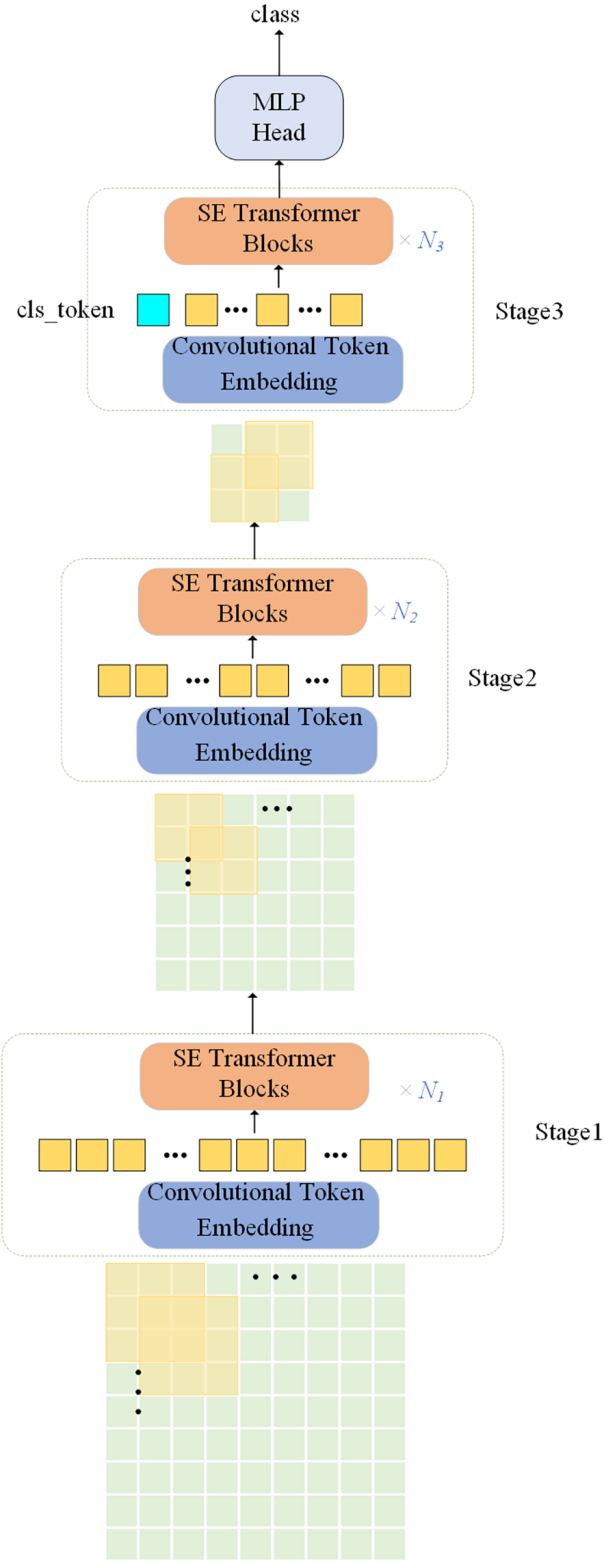
Overall architecture of the proposed SE-ViT model.

**Figure 2:**
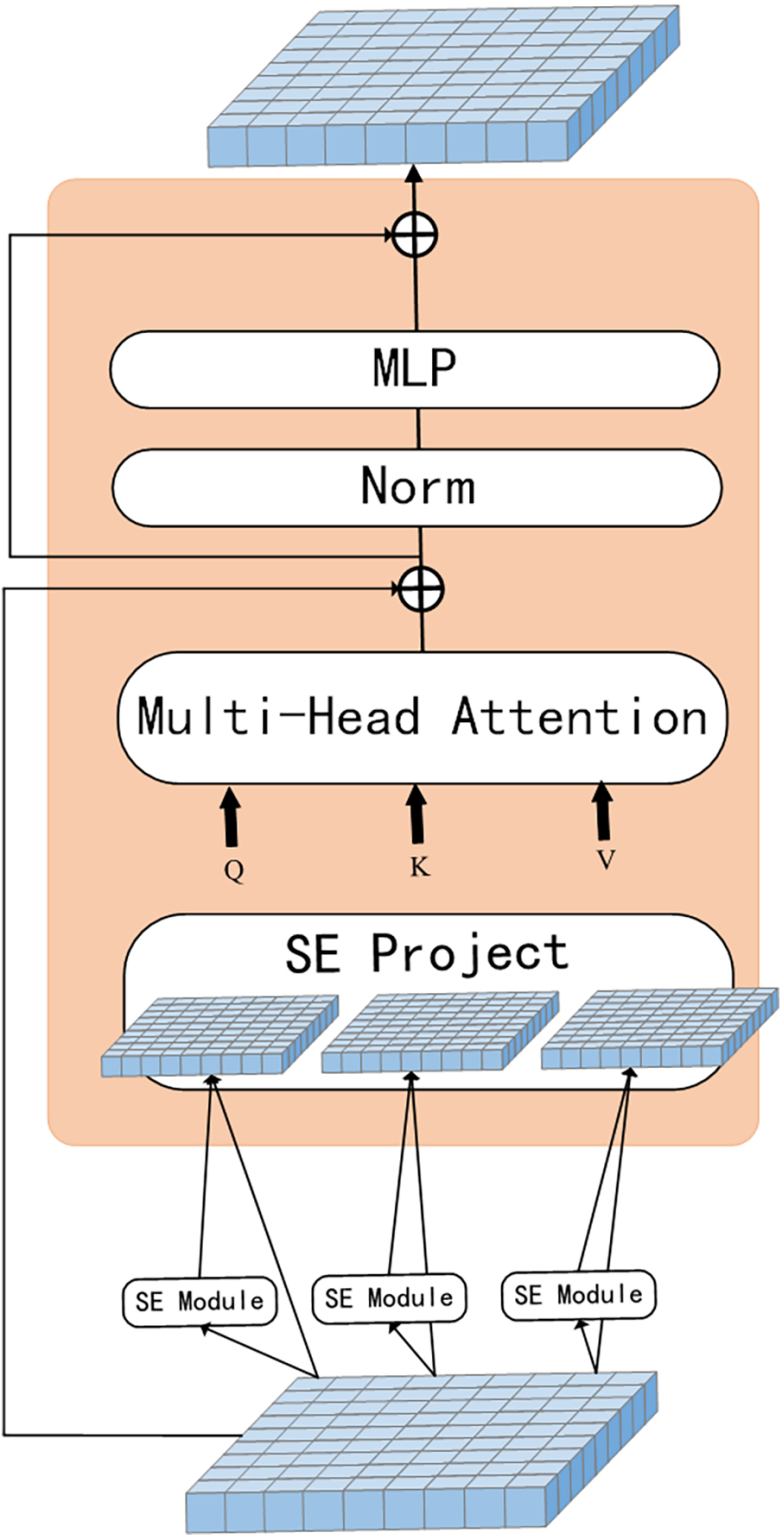
SE-Transformer block.

The proposed network model is illustrated in Fig. 1. Convolutional operation is introduced in the ViT model with the SE module, the model uses a multi-level hierarchy with three stages in total. Firstly, the input image is passed through the convolutional token embedding layer, then into the normalization layer. The advantage of this structure is that the feature resolution of the tokens can be gradually reduced while increasing the feature dimension of the tokens, achieving spatial downsampling and adding a rich feature representation. Figure 2 illustrates the SE Transformer module. It effectively highlights important features and reduces the impact of unimportant features for computing Query, Key and Value values. [CLS] classification tokens are added in the third stage. Finally, the final classifications are predicted using the MLP head.

### Convolutional token embedding

This module uses a convolution operation to extract local features from the input feature map. For the 2D image generated in the previous stage *x*_*i*−1_ ∈ ℝ^*H*_^*i*−1^_×*W*_^*i*−1^_×*C*_^*i*−1^_^, it is mapped into a new tokens *f* (x_*i*−1_) using the function *f* (⋅), which is sent as input to the next stage *i*. Where *f* (⋅) is a 2D convolution operation with convolution kernel size *s* ×* s*, step size *s-o* and *p* is the size of the padding. Hence, the height and width of token *f*(*x*_*i*−1_) ∈ ℝ^*H*_^*i*^_×*W*_*i*_×*C*_^*i*^_^ are calculated as shown in Eq. (1):

(1) \begin{eqnarray*}{H}_{i}= \left\lfloor \frac{{H}_{i-1}+2p-s}{s-o} +1 \right\rfloor ,{W}_{i}= \left\lfloor \frac{{W}_{i-1}+2p-s}{s-o} +1 \right\rfloor \end{eqnarray*}

Then, *f* (x_*i*−1_) is flattened to a shape of size *H*_*i*_ *W*_*i*_ × *C*_*i*_ and fed into the subsequent transformer blocks at stage *i*.

Convolutional token embedding adjusts the feature dimension of the tokens and the number of tokens at each stage by means of convolutional operation. In this way, the length of the tokens sequence is gradually reduced at each stage while the feature dimension is increased. This gives tokens the ability to learn increasingly complex feature information on an increasingly large spatial scale.

### ResNet-SE-ViT

The CNN model achieves feature extraction by fusing the spatial and channel information of the image, with different convolutional kernels finding spatial features in each input channel. However, the CNN model cannot effectively highlight the important features when extracting features, as the network models have equal weights for each channel. To address this problem, the SE model is introduced in the attention mechanism.

**Figure 3:**
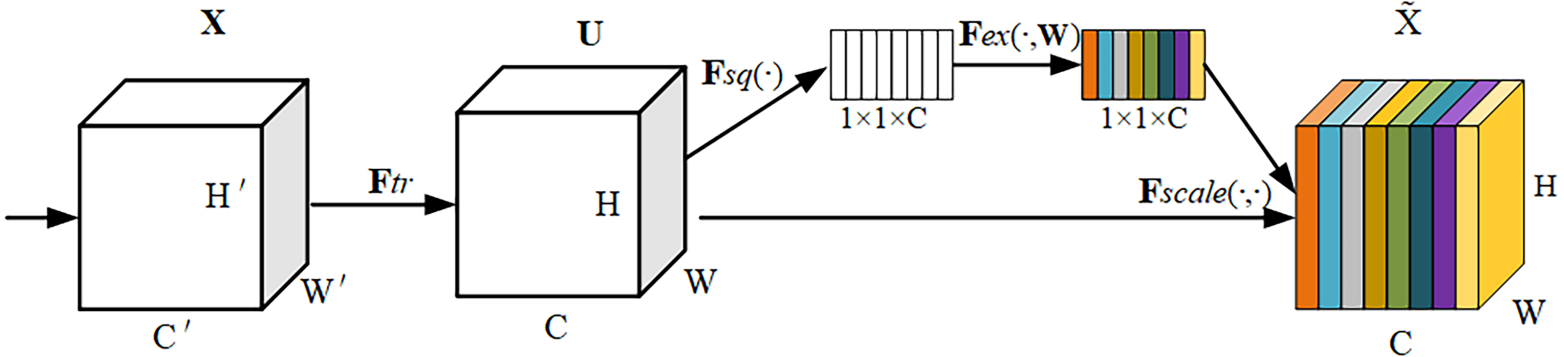
SE module structure.

The SE module adaptively weights each channel by adding a content-aware mechanism that compresses the feature maps to a single value so that the network model can adaptively adjust the weight of each feature map to obtain a global understanding of each channel. It has the simplicity and effectiveness to improve channel interdependencies at a small additional computational cost.The operational flow of the SE module is shown in Fig. 3.

The SE module consists of three components: Squeeze, Excitation and Scale. For an input feature map X with size *W*′ × *H*′ × *C*′, the feature map U is obtained by the convolution operation Ftr(⋅, *θ*) with size *W* × *H* × *C*The formula for Ftr is shown in Eq. (2).

(2) \begin{eqnarray*}{u}_{c}={v}_{c}\ast X=\sum _{s=1}^{{C}^{{^{\prime}}}}{v}_{c}^{s}\ast {X}^{s}\end{eqnarray*}

where vc denotes the c-th convolution kernel and X^s^ denotes the s-th input. Then after the SE module, weights are added to each channel of the feature map U to highlight important features to suppress redundant features.

**Squeeze:** For a feature map U of size *W* × *H* × *C*, a global average pooling is used to perform the squeeze operation on it, resulting in the output of a vector of size 1 × 1 × *C*, calculated as shown in Eq. (3).

(3) \begin{eqnarray*}{z}_{c}={F}_{sq}({U}_{c})= \frac{1}{H\times W} \sum _{i=1}^{H}\sum _{j=1}^{W}{u}_{c}(i,j).\end{eqnarray*}

After the Squeeze operation, each feature map can be effectively associated with other feature maps, increasing the global receptive field and extracting richer features, thus improving the accuracy of classification and recognition.

**Excitation:** To take advantage of the global information in the Squeeze operation, the Excitation operation captures the dependencies between each channel. Excitation consists of two fully connected layers with two activation functions, z is first multiplied by the first fully connected layer *W*_1_, where the dimension of z becomes $1\times 1\times \frac{C}{r} $. Then it passes through the ReLU activation function, which learns the nonlinear relationships of each channel. Then it passes through the second fully connected layer *W*_2_, where the dimension of z becomes 1 × 1 × *C*, is then passed through the sigmoid activation function to output the results. The formula is shown in Eq. (4):

(4) \begin{eqnarray*}s={F}_{ex}(z,W)=\sigma (g(z,W))=\sigma ({W}_{2}\delta ({W}_{1}z))\end{eqnarray*}

where ${W}_{1}\in {\mathbb{R}}^{ \frac{C}{r} \times C}$, *δ* is the ReLU activation function, ${W}_{2}\in {\mathbb{R}}^{C\times \frac{C}{r} }$ and *σ* is the sigmoid activation function.

**Scale:** Using the weights learned by Excitation to scale U, the weights of each channel are multiplied with the matrix of the corresponding channel of U respectively, and finally the feature map with the weight information is obtained. The calculation formula is shown in Eq. (5):

(5) \begin{eqnarray*}\widetilde {{X}_{C}}={F}_{scale}({u}_{c},{s}_{c})={s}_{c}\cdot {u}_{c}.\end{eqnarray*}

The SE module highlights feature maps with large weight values and ignores those with invalid or small weight values. At the same time, the inclusion of the SE module inevitably increases the number of parameters and computations, but these effects are acceptable in terms of improving performance.

**SE-ViT:** Fig. 2 illustrates the process of capturing global features by the SE module. Tokens are first reshaped as a 2D token map and next the Q, K and V values are calculated separately using the SE module, as shown in Eq. (6). Finally, the projected tokens are flattened as 1D vectors and fed as tokens into the next stage.

(6) \begin{eqnarray*}{x}_{i}^{q/k/v}=\text{Flatten}(\mathrm{SE}(\text{Reshape}2\mathrm{D}({x}_{i}))).\end{eqnarray*}

where ${x}_{i}^{q/k/v}$ is the *Q*/*K*/*V* matrix of the input token at layer *i* and x_*i*_ is the token that has not been extracted with features by the SE module.

### Position embedding

Position embedding is the key to learning semantic feature information of an image, which is robust to detailed texture variations of the image. However, Mao et al. (2021) pointed out that existing position embedding methods did not have much impact on the robustness of deep neural networks. By comparing four methods, namely learned absolute, sin-cos absolute, learned relative (Shaw, Uszkoreit & Vaswani, 2018) and input-conditioned (Chu et al., 2021), they found that the position embedding mechanism did not have a significant impact on improving the robustness of deep neural networks, in a few cases even decrease the robustness.

In this paper, the SE module is introduced for each Transformer block, which, in combination with convolutional token embedding, allows the network model to efficiently establish spatial relationships. Therefore, not adding position embedding to the neural network model does not reduce the robustness of the model, simplifying the design of the network model for vision tasks with different input resolutions.

## Experimental Design and Analysis of Results

### Experimental platform

The experimental platform for this study is based on ubuntu 18.04, with 128G of experimental running memory. Hardware equipment using a graphics card NVIDIA Tesla V100 GPU with 32G of video memory. The experimental environment uses the PyTorch deep learning framework that supports GPU accelerated computing, the cuda environment is configured with NVIDIA CUDA 11.3 and cuDNN V8.2.1 deep learning acceleration library.

### Dataset setup

This experiment uses the mini-ImageNet (Vinyals et al., 2016) dataset to verify the effectiveness of the model. mini-ImageNet contains a total of 100 categories, with 64 categories in the training set, 16 categories in the validation set, 20 categories in the test set, each containing 600 images, for a total of 60,000 data samples of size 84 × 84. During the experiments, the data are first preprocessed, the samples are upsampling to 299 × 299 pixel size, then the adversarial examples are generated using the white-box adversarial attack methods BIM, C&W, DeepFool, DI2FGSM, MDI2FGSM, the black-box adversarial attack methods P-RGF, RGF (Cheng et al., 2019) and Parsimonious (Moon, An & Song, 2019).

### Parameter setting

The parameters of the model were optimised during training using the AdamW (Loshchilov & Hutter, 2017) optimiser, with a learning rate set to *α* = 0.02, momentum set to 0.9 and a weight decay value of 0.05; the parameters were updated using the softmax loss function with a learning decay rate of 0.1. The parameter settings are shown in Table 1.

### Analysis of experimental results

#### Comparison with different network structures

The robustness of the proposed defense method against adversarial attacks is investigated and compared with different Transformer network structures such as TNT model, Pyramid TNT model, T2T model and DeiT model to test their accuracy against different adversarial attacks. The experimental results are shown in Table 2.

Table 2 shows the comparison between different Transformer network models and the ResNet-SE-ViT model. It can be seen that the accuracy of the proposed defense method can reach 18.985% in the face of MDI^2^FGSM attack with strong attack performance, while TNT-B is only 13.596%. The proposed defense method has high accuracy in the face of the adversarial attack and shows strong robustness, which is due to the fact that the proposed network model focuses on the content of the adversarial example, learning detailed features such as textures and lines in images, while extracting global features of the adversarial example.

**Figure 4:**
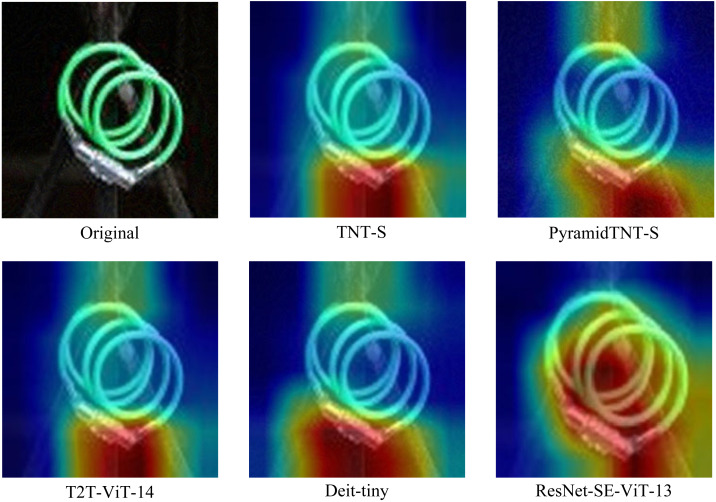
Comparison of different Transformer structures with ResNet-SE-ViT.

As can be seen from Fig. 4, the addition of the ResNet-SE module reinforces the feature information among channels and suppresses the secondary information, which helps the model to extract key features and further enhances the robustness of the network model.

**Table 1:** Training parameter settings.

Parameters	Values
Learning rate	0.02
Epoch	150
Weight decay	0.05
Batch size	64
Momentum	0.9
Learning rate decay	0.1

**Table 2:** Comparison of different Transformer network structures.

Method type		BIM	C&W	DeepFool	DI^2^FGSM	MDI^2^FGSM
Transformer	TNT-S	10.055	11.066	12.112	11.245	12.464
	TNT-B	11.060	13.562	11.083	11.914	13.596
	PyramidTNT-Ti	13.119	11.562	11.256	8.152	9.826
	PyramidTNT-S	14.551	12.179	11.943	10.654	10.288
	PyramidTNT-M	14.710	11.129	12.839	13.868	12.568
	PyramidTNT-B	15.146	12.156	13.303	12.685	12.260
	T2T-ViT-14	10.737	8.973	8.084	8.536	8.701
	T2T-ViT-19	12.685	10.195	9.187	9.344	9.312
	T2T-ViT-24	13.192	10.219	10.167	10.893	10.161
	DeiT-tiny	11.896	9.383	10.108	8.067	9.908
	DeiT-small	12.596	10.242	11.975	9.983	10.967
DeiT-base	13.781	11.325	10.458	10.867	9.042
ResNet-SE-ViT	ResNet-SE-ViT-13 ResNet-SE-ViT-13_384_ ResNet-SE-ViT-21 ResNet-SE-ViT-21_384_	13.684	16.260	18.490	21.146	16.100
		15.188	19.156	23.825	19.784	18.985
		19.812	17.083	18.802	21.490	18.010
		18.586	17.892	19.156	18.760	19.177

Compared with ResNet-SE-ViT-13, the other four methods cannot effectively capture the key features in the face of adversarial examples, such that the model misclassifies them; whereas the proposed defense method focuses on the core regions of the adversarial examples and extracts the key features. Therefore the robustness is stronger than the other models.

By comparing the four different structures in the ResNet-SE-ViT model, it is found that ResNet-SE-ViT-13 exhibits lower robustness compared to the other three structures. As the network model structure deepens, the robustness also increases, it is experimentally concluded that the resolution of the adversarial examples also affects the robustness of the network model, the adversarial examples with a resolution of 384 ×384 exhibits an overall better robustness than 224 ×224. Therefore, in the subsequent experiments, SE-ViT-21_384_ will be selected for comparison.

#### Comparison with CvT

The proposed defense method is an improvement of the convolutional vision Transformer (CvT) (Wu et al., 2021) method. The CvT model uses convolutional operations for mapping in the Transformer block. To verify the effectiveness of the ResNet-SE module, two model structures ResNet-SE-ViT and CvT are compared separately in terms of robustness in the face of white-box attacks. The experimental results are shown in Table 3.

As can be seen from Table 3, the accuracy of the proposed network structure ResNet-SE-ViT is higher than that of the CvT model in the face of adversarial examples, the accuracy of the proposed defense method is 21.490% in the face of DI^2^FGSM, while CvT_21_ is only 16.833%, which indicates that compared to using convolutional operations in the Transformer block, the ResNet-SE module can effectively highlight the important features of each channel and suppress the useless features, which helps the model to extract key features and enhance the robustness of the network model.

**Figure 5:**
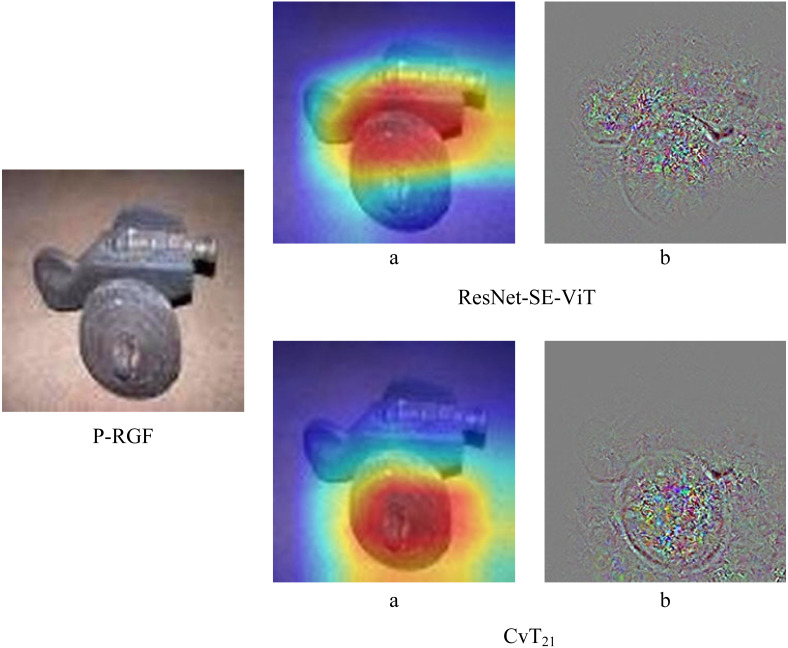
Differences between ResNet-SE-ViT and CvT when extracting features.

In order to further verify the robustness of the proposed method, the robustness of ResNet-SE-ViT and CvT in the face of black-box attacks are compared separately, experiments are conducted using three black-box attack methods, Parsimonious, P-RGF and RGF, respectively. The experimental results are shown in Table 4, it can be seen that the accuracy of the proposed defense methods are all higher than that of the CvT model. In addition, Fig. 5 shows the difference between ResNet-SE-ViT and CvT in the face of the black box attack P-RGF.

As can be seen from Table 4, the ResNet-SE-ViT model outperforms the CvT model in defending against the three black-box attacks, improving the accuracy of the adversarial examples by 4.534%, 3.914%, and 3.55%, respectively. Compared with CvT, Fig. 5 shows more intuitively that ResNet-SE-ViT focuses on key regions when extracting image features, focuses on the understanding of image content while paying attention to global information, further verifying that using the ResNet-SE module to replace the convolutional mapping in the CvT model can effectively enhance the robustness of the network model.

**Table 3:** Performance of ResNet-SE-ViT and CvT in defending against white-box attacks.

	ResNet-SE-ViT_21_	CvT_21_
BIM	19.812	17.958
C&W	17.083	15.785
DeepFool	18.802	17.208
DI^2^FGSM	21.490	16.833
MDI^2^FGSM	18.010	16.532

**Table 4:** Performance of ResNet -SE-ViT and CvT in defending against black box attacks.

	ResNet-SE-ViT	CvT_21_
Parsimonious	18.830	14.296
P-RGF	18.490	14.576
RGF	16.920	13.370

#### The role of the SE module

The SE module can effectively highlight the important features and reduce or suppress the unimportant ones. To verify the effectiveness of the SE module in the face of adversarial attacks, two different models of ResNet-ViT and ResNet-SE-ViT were experimented using a white-box attack approach. The experimental results are shown in Table 5.

**Table 5:** Performance of ResNet-SE-ViT and ResNet-ViT in the face of white-box attacks.

	ResNet-SE-ViT	ResNet-ViT	
BIM	19.812		18.080
C&W	17.083		15.890
DeepFool	18.802		14.260
DI^2^FGSM	21.490		19.370
MDI^2^FGSM	18.010		19.630

**Figure 6:**
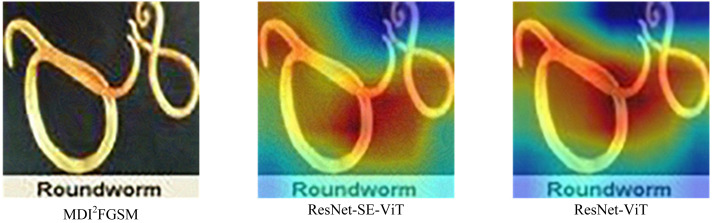
Differences in feature extraction between ResNet-SE-ViT and ResNet-ViT.

From Table 5, it can be concluded that compared with the ResNet-ViT network structure, the ResNet-SE-ViT structure shows higher accuracy in the five adversarial attack methods, for example, in the DI^2^FGSM attack method, the accuracy of ResNet-SE-ViT is 2.120% higher than that of ResNet-ViT, which indicates that the SE module plays an important role, which can effectively highlight the key features of the adversarial example, suppressing the perturbations in the adversarial example and mitigate the impact of the perturbations on the network structure as a way to enhance the robustness of the network structure; however, in the face of MDI^2^FGSM attack method, the defense performance of ResNet-SE-ViT is slightly lower than that of ResNet-ViT. By comparing the two models for analysis in Fig. 6, it is found that compared to ResNet-ViT, ResNet-SE-ViT does not extract the key feature regions of the image in the face of MDI^2^FGSM attack method, ResNet-SE-ViT does not focus on the key features in extracting the features, which leads to making wrong judgments in the final classification, so the performance is lower than that of ResNet-ViT.

To further verify the effectiveness of the SE module, experiments are conducted on ResNet-SE-ViT and ResNet-ViT using different black-box attack methods, the experimental results are shown in Table 6.

**Table 6:** Performance of ResNet-SE-ViT and ResNet-ViT in the face of black-box attacks.

	ResNet-SE-ViT		ResNet-ViT
Parsimonious	18.830	14.480	
P-RGF	18.490	14.020	
RGF	16.920	15.040	

From Table 6, it can be concluded that the accuracy of ResNet-SE-ViT model is 1.880% higher than that of ResNet-ViT in the face of RGF adversarial attack algorithm, the accuracy of ResNet-SE-ViT is 4.350% and 4.470% higher than the ResNet-ViT model in the face of Parsimonious and P-RGF black box attacks, respectively, showing strong robustness, which further illustrates that adding SE module to the network structure can effectively defend against both white-box and black-box attacks and improve the robustness of the network model.

**Figure 7:**
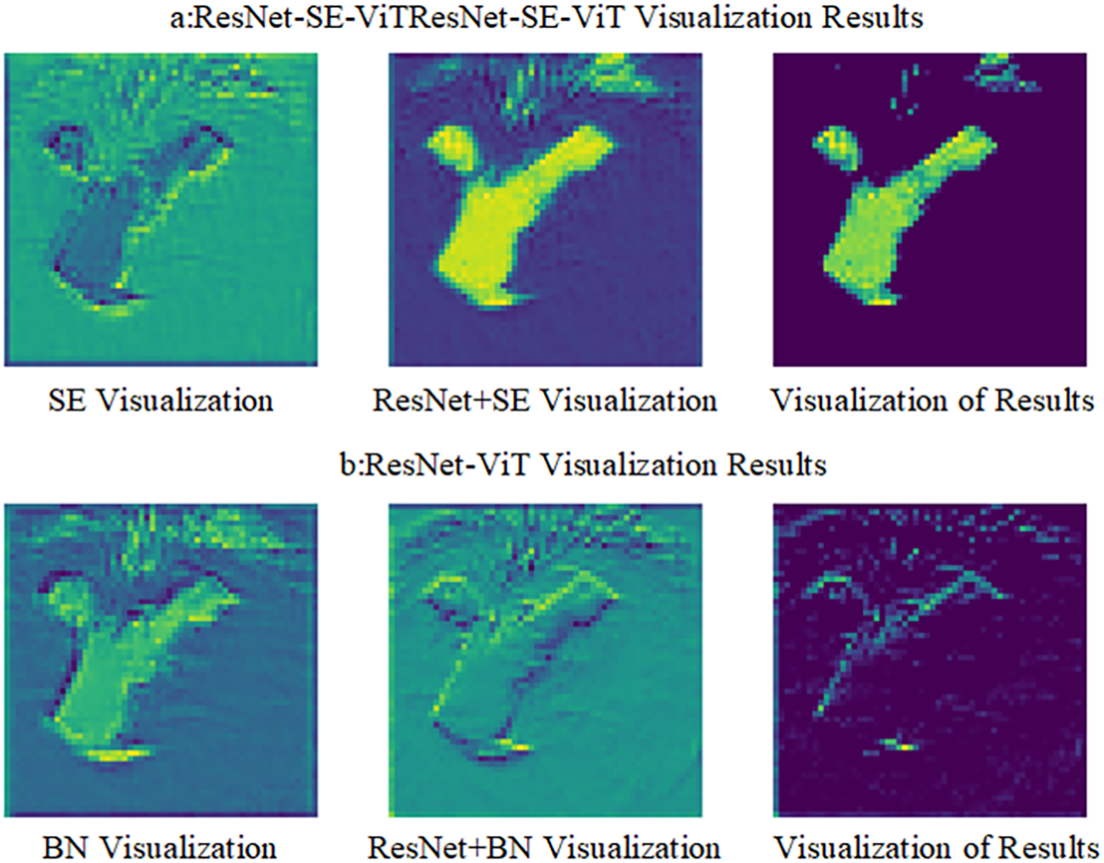
Visualization of two different defense methods.

To visualize the effect of the SE module in the defense method, the two defense methods are visualized by comparing the differences in feature extraction between the ResNet-SE-ViT and ResNet-ViT models, the results are shown in Fig. 7, where SE viewable represents the result after the SE module, x represents the input data of the ResNet network, BN viewable represents the result after the batch normalization layer, output indicates the result after the activation function ReLU.

Figure 7A shows the viewable view of features extracted by the ResNet-SE-ViT defense method, Fig. 7B shows the viewable view of the ResNet-ViT defense method. Comparing the results of the two models in the viewable view reveals that the SE module can effectively highlight the content information of the image, focusing on the image feature regions and less on the high frequency information of the image. It indicates that the addition of the SE module will help the model defend against the impact of adversarial attacks and improve the robustness of the model itself.

#### Ablation experiments

In order to study the rationality of the proposed defense method, different ablation experiments are designed. First, the role of cls_token in different stages is investigated; then, the effect of different number of transformer blocks in different stages on the robustness of the neural network is studied; finally, the effect of position embedding in different positions is analyzed.

**CLS_Token:** ResNet-SE-ViT model adds cls_token in the third stage, to investigate the effect of cls_token on the robustness of the network structure at different stages, it is validated on the dataset DI^2^FGSM, the experimental results are shown in Table 7, where d indicates that cls_token is not used in the network model.

**Table 7:** Performance of cls_token in different locations.

	Stage 1	Stage 2	Stage 3	Rob.Acc
a	✓			16.530
b		✓		20.010
c			✓	21.490
d				20.820

From Table 7, it can be seen that adding CLS_Token in the third stage can effectively improve the robustness of the network model compared to a and b adding CLS_Token in the first and second stages, the accuracy can reach 21.490%, there is also an improvement in robustness compared to d not using CLS_Token, indicating the rationality of the proposed defense method. The reason for this result is that if the CLS_Token is used too early in the first and second stages, the token will follow the subsequent network model for training, integrating visual features at different locations, but these features contain perturbations that have been added, which will eventually affect the robustness of the neural network.

**Transformer Blocks:** To research the effect of the number of Transformer blocks on the robustness of the ResNet-SE-ViT model at different stages, different numbers of blocks are set at each stage and the total number of Transformer blocks is kept as 21, the experimental results are shown in Table 8, where Mem denotes memory consumption.

From Table 8, it can be concluded that setting different numbers of Transformer blocks at different stages makes a difference in the robustness of the neural network. For example, compared to methods b and c, when the number of Transformer blocks in method a is set to 16, the network model shows a better robustness accuracy of 21.490%, but with a larger memory consumption of 115.4 M. By comparing the a, b, c and d methods, it is found that the robustness of the model is improved when the third stage of the model contains more Transformer blocks with large spatial resolution; on the contrary, the robustness and memory consumption of the model decrease when the number of Transformer blocks is gradually reduced. The a method is chosen as the superior method under the comprehensive consideration of the model robustness performance.

**Figure 8:**
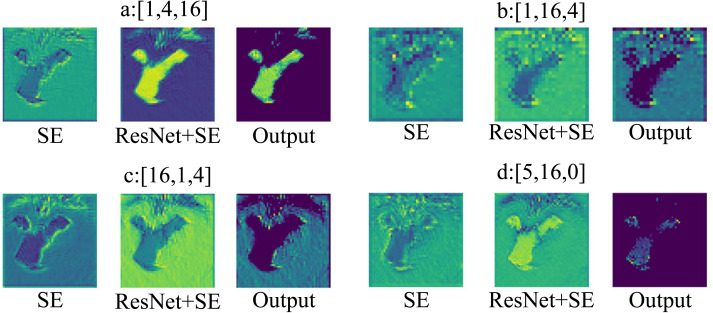
Transformer blocks at different stages.

Similarly, to better demonstrate the differences in the number of Transformer blocks in extracting features at different stages, the results of different layers are visualized as shown in Fig. 8. Figures 8A, 8B, 8C, 8D, represent the visualizable views of the different layers in the three stages, respectively. As in Fig. 8A, SE denotes the visualization result after the SE module, x+SE denotes the output result of the SE module summed with the input data of the ResNet network, and Output is the result after the activation function ReLU.

**Table 8:** Effect of the number of transformer blocks on robustness at different stages.

	[S1,S2,S3]	Mem	Rob.Acc
a	[1,4,16]	115.4M	21.490
b	[1,16,4]	55.1M	20.594
c	[16,1,4]	32.7M	17.510
d	[5,16,0]	29.0M	14.560

Figure 8A shows that when stage S3 has more Transformer blocks, ResNet-SE-ViT can focus on the content information when extracting features; From Figs. 8B and 8C, it can be seen that when stages S1 and S2 have more Transformer blocks, the network model does not focus on the content information of the images, and will add noise and other unimportant information when extracting features, which is not conducive to improving the robustness of the network model; Fig. 8D indicates that Transformer blocks are not set in stage S3, the content information exhibited by the images is not obvious, resulting in the network model not being able to fully extract the image information, so that the model has poor robustness. Therefore, with the consideration of model robustness performance, method a is chosen as the better method in this work.

**Position Embedding:** The position embedding encodes the position of each token, which is crucial for learning shape-based semantic features and is robust to texture changes. To research the effect of position embedding on the ResNet-SE-ViT model, position embedding is added at different stages. The experimental results are shown in Table 9, where d indicates that position embedding is not used in the model.

**Table 9:** Effect of position embedding on model robustness.

	Stage 1	Stage 2	Stage 3	Rob.Acc
a	✓			18.680
b		✓		19.730
c			✓	18.310
d				21.490

By comparing the three methods a, b and c, it is found that the different stages of position embedding do not have much effect on the robustness of the network model with the accuracy of 18.680%, 19.730% and 18.310%, respectively, while the accuracy of method d increases after removing the position embedding, which indicates that the position embedding is easy to change with the change of the input, if the appropriate position embedding method is not chosen, it will cause the robustness of the network model to become worse. Therefore, the proposed defense method in this paper does not use position embedding, uses SE module and convolutional token embedding to make the model establish the position relationship between image blocks.

## Conclusion

In this work, an effective defense method ResNet-SE-ViT is proposed by introducing ResNet structure and SE module. Firstly, the ViT model is slightly more robust than the CNN model; secondly, the ViT model can effectively extract the global information of features and capture the global similarity of features, while the SE module focuses on the detailed information of images such as textures and lines, highlighting the key information of feature maps and suppressing the secondary information. The introduction of convolution operation in ViT helps the model to extract increasingly complex feature information. The results show that the proposed defense method can effectively defend against both white-box and black-box attacks with strong robustness.

In the adversarial example defense task, we propose an effective defense method that is more accurate than other ViT models, but still less accurate compared to CNN models. Therefore, in future work, the robustness of ViT models can be further improved by drawing on the adversarial training method.

##  Supplemental Information

10.7717/peerj-cs.1197/supp-1 Supplemental Information 1 Code

## Additional Information and Declarations

### Data Availability

The following information was supplied regarding data availability: The data and the code are available in the Supplemental Files.
